# *Trichoderma atroviride* LZ42 releases volatile organic compounds promoting plant growth and suppressing Fusarium wilt disease in tomato seedlings

**DOI:** 10.1186/s12866-022-02511-3

**Published:** 2022-04-05

**Authors:** Yuxin Rao, Linzhou Zeng, Hong Jiang, Li Mei, Yongjun Wang

**Affiliations:** 1 College of Forestry and Biotechnology, Zhejiang Agricultural and Forestry University, Hangzhou, 311300 China; 2 State Key Laboratory of Subtropical Silviculture, Zhejiang Agricultural and Forestry University, Hangzhou, 311300 China

**Keywords:** Biostimulation, Biocontrol, 6-pentyl-2H-pyran-2-one, Inhibitory effect

## Abstract

**Background:**

The promotion of plant growth and suppression of plant disease using beneficial microorganisms is considered an alternative to the application of chemical fertilizers or pesticides in the field.

**Results:**

A coconut-scented antagonistic *Trichoderma strain* LZ42, previously isolated from *Ganoderma lucidum*-cultivated soil, was investigated for biostimulatory and biocontrol functions in tomato seedlings. Morphological and phylogenetic analyses suggested that strain LZ42 is closely related to *T. atroviride*. Tomato seedlings showed increased aerial and root dry weights in greenhouse trials after treatment with *T. atroviride* LZ42 formulated in talc, indicating the biostimulatory function of this fungus. *T. atroviride* LZ42 effectively suppressed Fusarium wilt disease in tomato seedlings, with an 82.69% control efficiency, which is similar to that of the carbendazim treatment. The volatile organic compounds (VOCs) emitted by *T. atroviride* LZ42 were found to affect the primary root growth direction and promote the root growth of tomato seedlings in root Y-tube olfactometer assays. The fungal VOCs from *T. atroviride* LZ42 were observed to significantly inhibit *F. oxysporum* in a sandwiched Petri dish assay. SPME–GC–MS analysis revealed several VOCs emitted by *T. atroviride* LZ42; the dominant compound was tentatively identified as 6-pentyl-2H-pyran-2-one (6-PP). The VOC 6-PP exhibited a stronger ability to influence the direction of the primary roots of tomato seedlings but not the length of the primary roots. The inhibitory effect of 6-PP on *F. oxysporum* was the highest among the tested pure VOCs, showing a 50% effective concentration (EC_50_) of 5.76 μL mL^−1^ headspace.

**Conclusions:**

*Trichoderma atroviride* LZ42, which emits VOCs with multiple functions, is a promising agent for the biostimulation of vegetable plants and integrated management of Fusarium wilt disease.

**Supplementary Information:**

The online version contains supplementary material available at 10.1186/s12866-022-02511-3.

## Background

Members of *Trichoderma* spp. are ubiquitous in the soil and wood as soil inhabitants, plant symbionts, and mycoparasites [1]. Certain species of this genus are the most versatile biofertilizer and/or biocontrol agents and are presently used as active ingredients in biopesticides, biofertilizers, growth enhancers, and stimulants of natural resistance [2, 3]. Over 60% of all the biopesticides registered in China contain a single *Trichoderma* isolate or mixtures of *Trichoderma* species (http://www.chinapesticide.org.cn/sjzx4ywb/index.jhtml, August 4, 2021). The well-known species that have been commercially developed include *T. harzianum*, *T. virens*, *T. viride*, *T. asperellum*, and *T. atroviride* [4]. These fungi are considered safe for human and livestock, and beneficial for crop plants through colonizing plant roots, without apparent adverse responses [5].

In recent decades, the multiple biological functions of *Trichoderma*, including the suppression of plant disease, the promotion of plant growth, decomposition, and bioremediation, have been well-documented. *Trichoderma* spp. have been reported to exhibit antagonistic activity against a broad spectrum of plant pathogenic fungi and oomycetes, such as *Fusarium oxysporum* [6, 7], *Phytophthora colocasiae* [8], and *Pythium graminicola *[9], as well as plant pathogenic bacteria [10] and nematodes [11]. The many secondary metabolites produced by *Trichoderma* species are responsible for their highly antagonistic effects, contributing to the success of their application [12]. Additionally, the application of *Trichoderma* species successfully regulates root architecture, increasing the length of lateral and primary roots, leading to the more efficient uptake of nutrients by the plant, resulting in biostimulation [13–15]. The mechanism through which *Trichoderma* exerts biostimulatory effects involves multilevel communication with root and shoot systems, including releasing many active metabolites into the rhizosphere, thereby increasing plant growth and yield [2].

Among the diverse secondary metabolites produced by *Trichoderma* species, a group of gas-phase, carbon-based compounds that are able to diffuse through the atmosphere and soils, called volatile organic compounds (VOCs), exhibit multiple biological functions [16–18]. The VOCs detected for *Trichoderma* comprise simple hydrocarbons, heterocycles, aldehydes, ketones, alcohols, phenols, thioalcohols, thioesters, and their derivatives [19]. Over 500 different VOCs in *Trichoderma* species had been detected according to a database of microbial volatiles (mVOC database) [20]. Some of these VOCs were reported to induce plant resistance or directly promote plant growth [21–23], and some were shown to be detrimental to plant pathogens [24, 25]. These findings indicate that VOCs may play a role in the biostimulatory and biocontrol activities of *Trichoderma* spp. [13, 17, 26].

Our previous study demonstrated the abundance of antagonistic *Trichoderma* species growing on the continuously cultivated soil surface of *Ganoderma lucidum*, an important Chinese traditional medicinal mushroom with high economic value [27]. Regardless of the continuous cultivation of the mushroom leading to problems due to increased populations of *Trichoderma*, the diverse species of *Trichoderma* in mushroom houses might provide bioresources for biostimulation and biocontrol in vegetable crop production. In this study, three *Trichoderma* strains with strong coconut-like scents were identified and investigated as potential biocontrol agents and plant stimulants. In addition, the emitted VOCs were analyzed; the antagonistic activity and effects on root growth of tomato seedlings of the VOCs were determined.

## Results

### Identification and morphological characterization of the scented strain LZ42

**Fig. 1:**
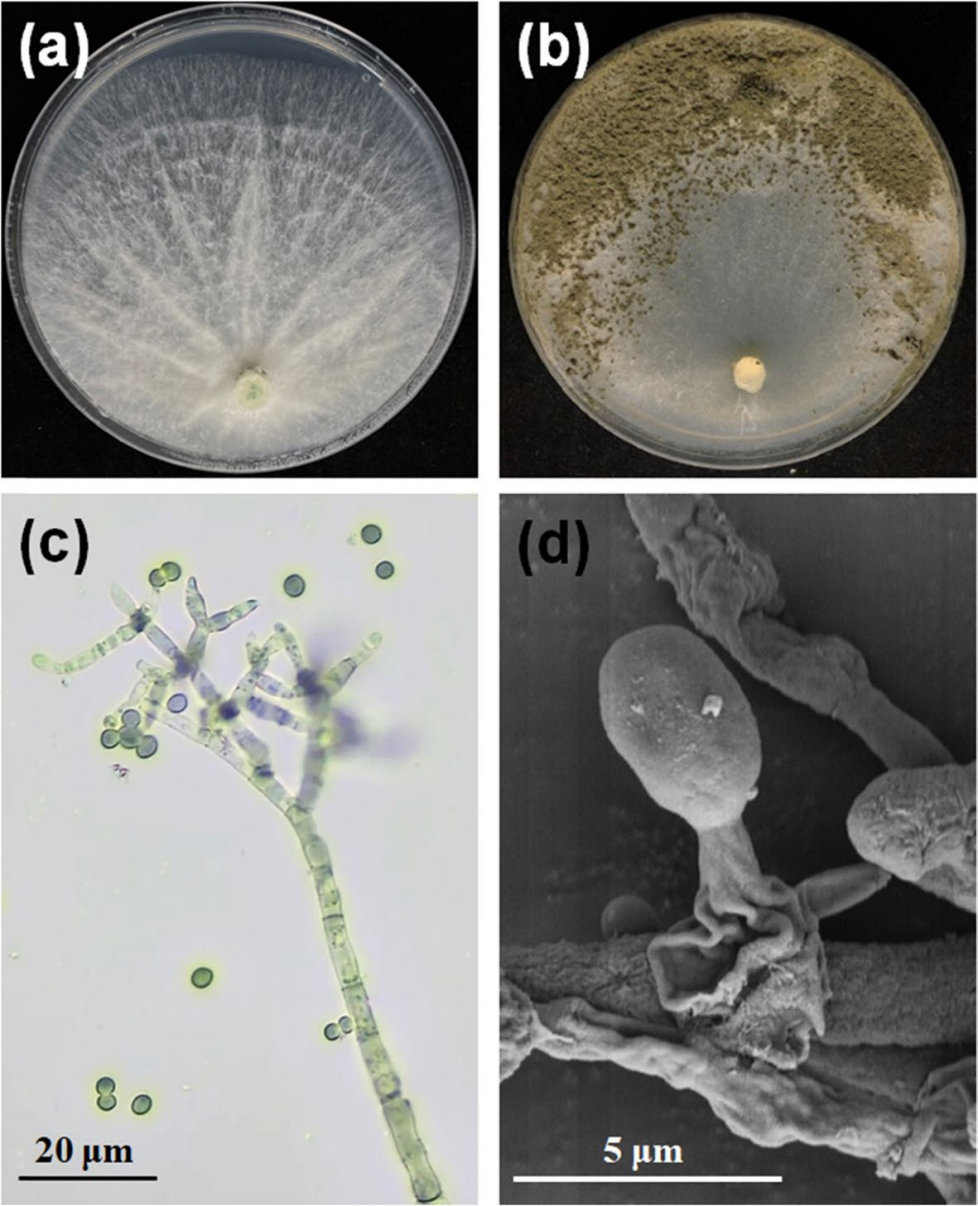
Morphological characteristics of *Trichoderma atroviride* LZ42. **a** Colony of *T. atroviride* LZ42 grown for 3 days on PDA. **b** Colony of *T. atroviride* LZ42 grown for 14 days on CMD. **c** Conidiophores and conidia formed on CMD under optical microscopy. **d** Conidiophores and conidia formed on CMD under SEM

**Fig. 2:**
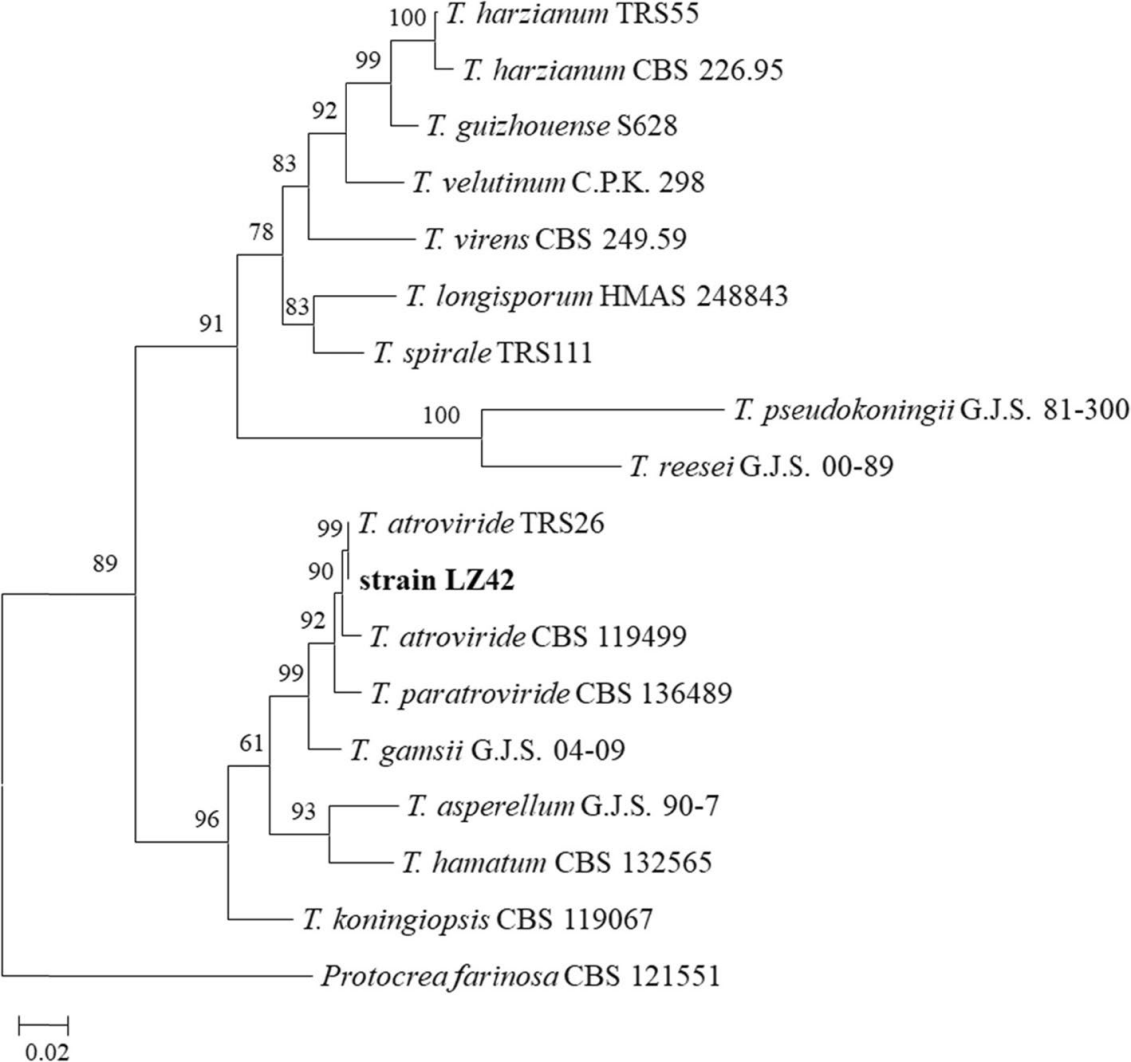
Phylogenetic tree generated from the combined sequences of *rpb2* and *tef1* loci of the genus *Trichoderma* spp. The tree was formulated using Mega 7.0 with 1000 bootstrap replications, condensed with a 70% cut-off value

During the isolation and culture of the *Trichoderma* isolates from soil under continuous *G. lucidum* cultivation in China, three of the total of 214 isolates emitted a strong coconut-like scent when fungi grew on potato dextrose agar (PDA) plates 5 days after inoculation. These three isolates exhibited the same ITS sequences and the same colony morphological characteristics (data not shown). Isolate LZ42 was selected for further identification and research as the representative strain. LZ42 grown on a PDA plate for 3 days exhibited a well-defined hyaline colony with typical aerial hyphae (Fig. 1a). Strong coconut-like odors were released when the plate was opened. The fungal conidiation started after 7 days, followed by a change from white to dark green in the outer half of the colony (Fig. 1b). The conidiophores emerged radially from the slender reticulum, consisting of a main axis and often distantly spaced side branches (Fig. 1c). The phialides formed on a widened supporting cell at the end of the conidiophores. The conidia were sub-globose to globose, smooth, and thick-walled (Fig. 1d). The characterization of the combinations of *rdp2* and *tef1* revealed the identity of strain LZ42 to be *T. atroviride*. Phylogenetic analysis based on the combinations of the *rdp2* and *tef1* gene sequences of different *Trichoderma* spp. revealed strain LZ42 to be more closely related to *T. atroviride* in the neighbor joining tree (Fig. 2).

### The effect of *Trichoderma atroviride* LZ42 formulation on the plant vegetable growth and control of Fusarium wilt disease

**Fig. 3:**
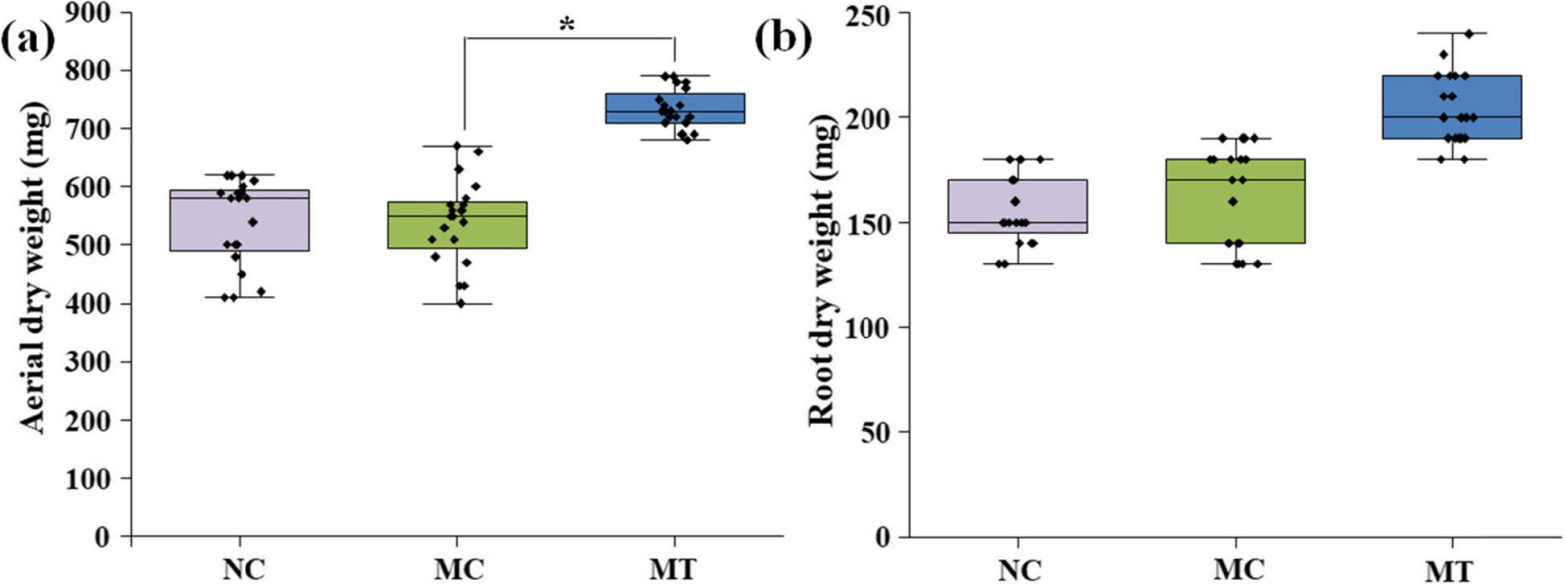
Effects of *Trichoderma atroviride* LZ42 on the phenotypic characteristics of aerial dry weight **(a)** and root dry weight **(b)** of tomato seedlings. The box indicate the range of the central 50% of the data from each treatment. The central lines mark the median value. The error bars across the boxes indicate the standard errors. Asterisks (*) in the figure indicate significant differences derived from two-sample comparisons (*P* = 0.05). NC indicates the treatment with uninfested soil. MC indicates the treatment amended with 50 g of the talc formulation alone. MT indicates the treatment amended with 50 g of the strain LZ42 formulation

At 15 days post-inoculation with *T. atroviride* LZ42 formulated in talc, the plant growth of the tomatoes was significantly promoted, as reflected in the increases in the aerial and root dry weights of the total tomato plants (Fig. 3). The mean aerial and root dry weights of the tomatoes treated with *T. atroviride* LZ42 were 742.36 mg and 209.64 mg, respectively, which were increases of 37.03% and 31.25% compared to the those for the treatment with talc alone (541.75 mg and 159.73 g), respectively. We observed no differences between the negative control and treatment with talc alone.

The incidence of tomato Fusarium wilt disease after 14 dpi is summarized in Table 1. The fungal pathogen *F. oxysporum* K34 exhibited high virulence to tomato seedlings in the greenhouse, with a 59.67% disease incidence. We found significant (*P* = 0.05) differences between the FT (10.25%) and FI treatments (59.67%) in disease incidence. The control efficiency of the *T. atroviride* LZ42 formulation was 82.69%. No significant differences between the FT and FC treatments were observed, indicating that *T. atroviride* LZ42 reduced the disease incidence with an efficiency equal to that of carbendazim.

**Table 1:** Efficacy of *Trichoderma atroviride* strain LZ42 and carbendazim in control of Fusarium wilt on tomato seedlings

Treatment^†^	% Disease incidence	% Control efficiency
FA	59.67 ± 3.21 ^b^	‒
FT	10.33 ± 1.53 ^a^	82.69
FC	7.67 ± 1.15 ^a^	87.15

^**†**^FA indicates the treatment with Fusarium-infested soil amended with 50 g of the talc formulation alone. FT indicates the treatment with Fusarium-infested soil amended with 50 g of the strain LZ42 formulation. FC indicates the treatment with Fusarium-infested soil mixed with carbendazim. Values are means of 3 replicates ± standard errors (SEs). Different superscripted lowercase letters indicate values that are significantly different (*P* = 0.05) within the same columns

### Effects of VOCs produced by *Trichoderma atroviride* LZ42 on root growth of tomato seedlings in Y-tube olfactometers

**Fig. 4:**
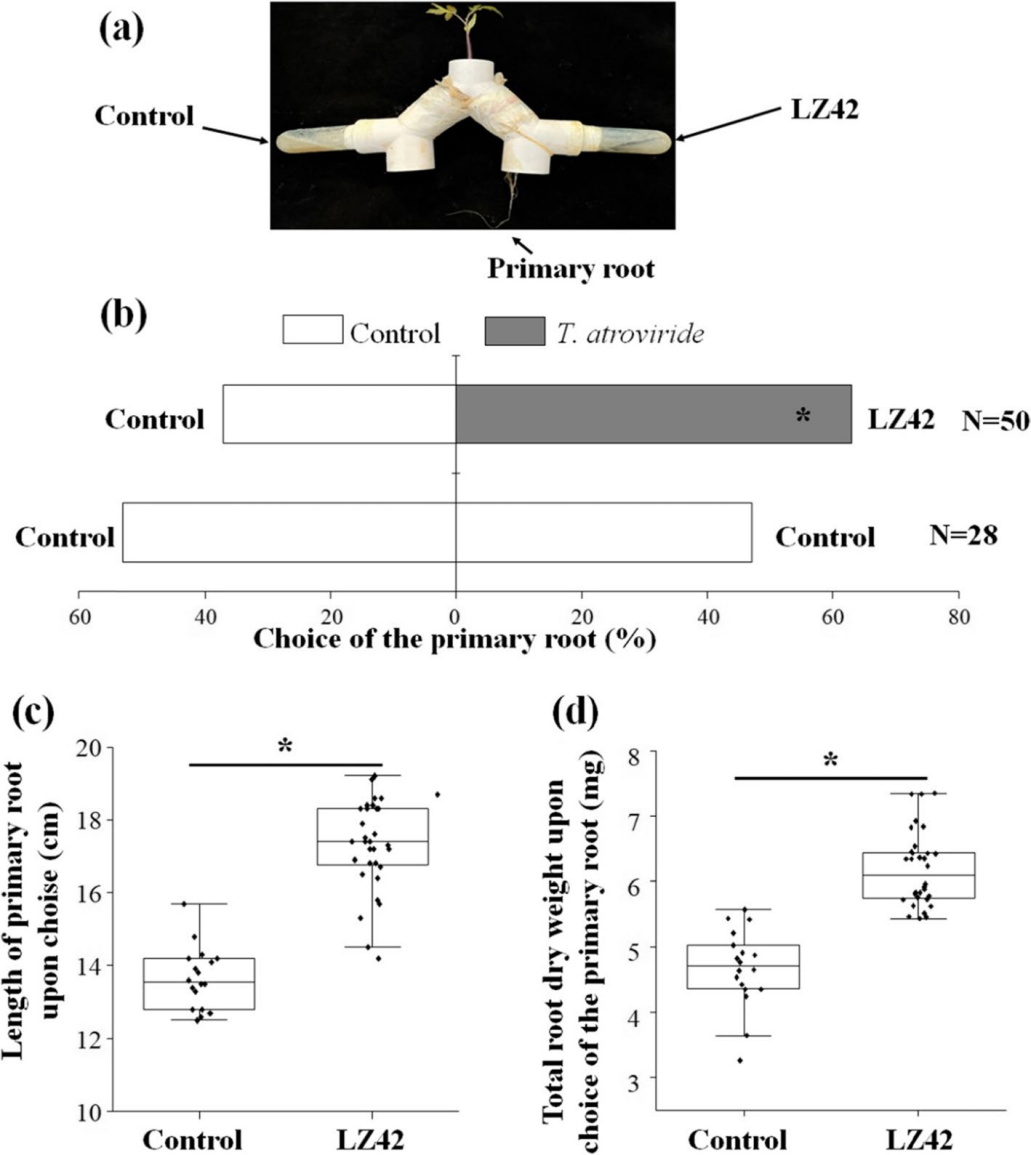
Effects of the VOCs produced by *Trichoderma atroviride* LZ42 on the root growth of tomato seedlings in modified root Y-tube olfactometers. **a** Tomato seedlings grown in the root Y-tube olfactometers for 14 days. **b** Percentages of tomato primary roots growing in the control arm or the LZ42 arm 14 days after sowing. Control versus control was used as a negative control. N indicates the number of root olfactometer replicates. Choices of primary roots were analysed with binomial tests (H_0_ = 0.50). Asterisk indicates pairwise differences between the control and LZ42 choices. **c** Length of the primary root. **d** Total root dry weight of the primary root. Asterisks (*) in the figure indicate significant differences derived from two-sample comparisons (*P* = 0.05)

The effects of the volatiles produced by *T. atroviride* LZ42 on the directional growth and growth rate of tomato seedling roots were tested in Y-tube olfactometers (Fig. 4a). Primary roots grew more frequently in the arm toward volatiles from *T. atroviride* LZ42 than in the arm toward volatiles from the control (‘choice’ = 63.33%; *P* = 0.50) (Fig. 4b). The means of the length of the primary roots and total root dry weight with treatment with VOCs from *T. atroviride* LZ42 were 17.31 cm and 5.88 mg, respectively, which are higher than those of the control (13.65 cm and 4.67 mg, respectively; *P* = 0.05) (Fig. 4c and Fig. 4d).

### Antifungal activity of VOCs produced by *Trichoderma atroviride* LZ42

**Fig. 5:**
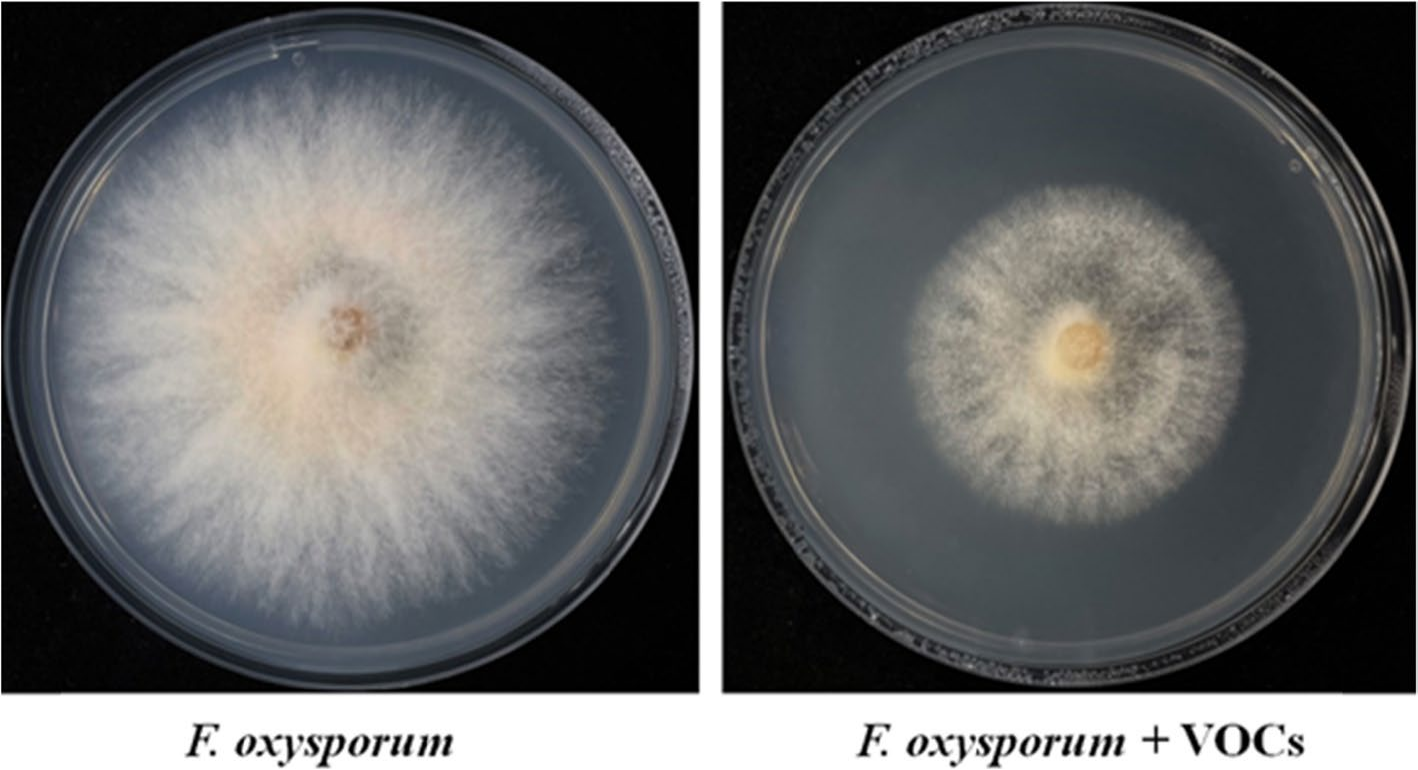
Antifungal activity of the volatile organic compounds emitted by *Trichoderma atroviride* LZ42 on the mycelial growth of *Fusarium oxysporum* f. sp. *lycopersici* K34 after sandwiched Petri plates assay

To determine the effects of the VOCs emitted by *T. atroviride* LZ42 on the mycelial growth of the plant pathogen, a volatile antifungal assay was conducted. We found that the colony diameters of the *F. oxysporum* treated with the VOCs of *T. atroviride* LZ42 were smaller than those of the control (Fig. 5). The percentage inhibition of *F. oxysporum* by the volatiles from *T. atroviride* LZ42 was 54.57%.

### Identification of VOCs through SPME GC/MS

Based on the SPME GC/MS analysis and comparison of the mass spectrum in the GC–MS system NIST data bank, the VOCs produced by *T. atroviride* LZ42 on PDA medium were tentatively identified with as five compounds based on similarities greater than 90%: 2-heptanone, 2-pentyl furan (2-PF), β-phellandrene, 3,5,5-trimethyl-cyclohexene, and 6-pentyl-2H-pyran-2-one (6-PP) (Table 2). The data from the relative area of each peak showed that 6-pentyl-2H-pyran-2-one was the most abundant compound emitted by *T. atroviride* LZ42, followed by 2-pentyl furan and β-phellandrene. The mass spectrum of the five compounds produced by *T. atroviride* LZ42 were shown in the Supplementary Fig. S1.

**Table 2:** The volatile compounds produced by *Trichoderma atroviride* LZ42 tentatively identified through SPME GC/MS analysis

RT (min)	Volatile compound	Formula	Similarity	% Area
12.25	2-heptanone	C_7_H_14_O	92	2.74
13.13	2-pentylfuran	C_9_H_14_O	93	3.54
14.28	β-phellandrene	C_10_H_16_	89	3.02
18.39	3,5,5-trimethyl-cyclohexene	C_9_H_16_	90	2.28
28.14	6-pentyl-2H-pyran-2-one	C_10_H_14_O_2_	94	48.26

### Effects of pure VOCs on root growth of tomato seedlings

**Fig. 6:**
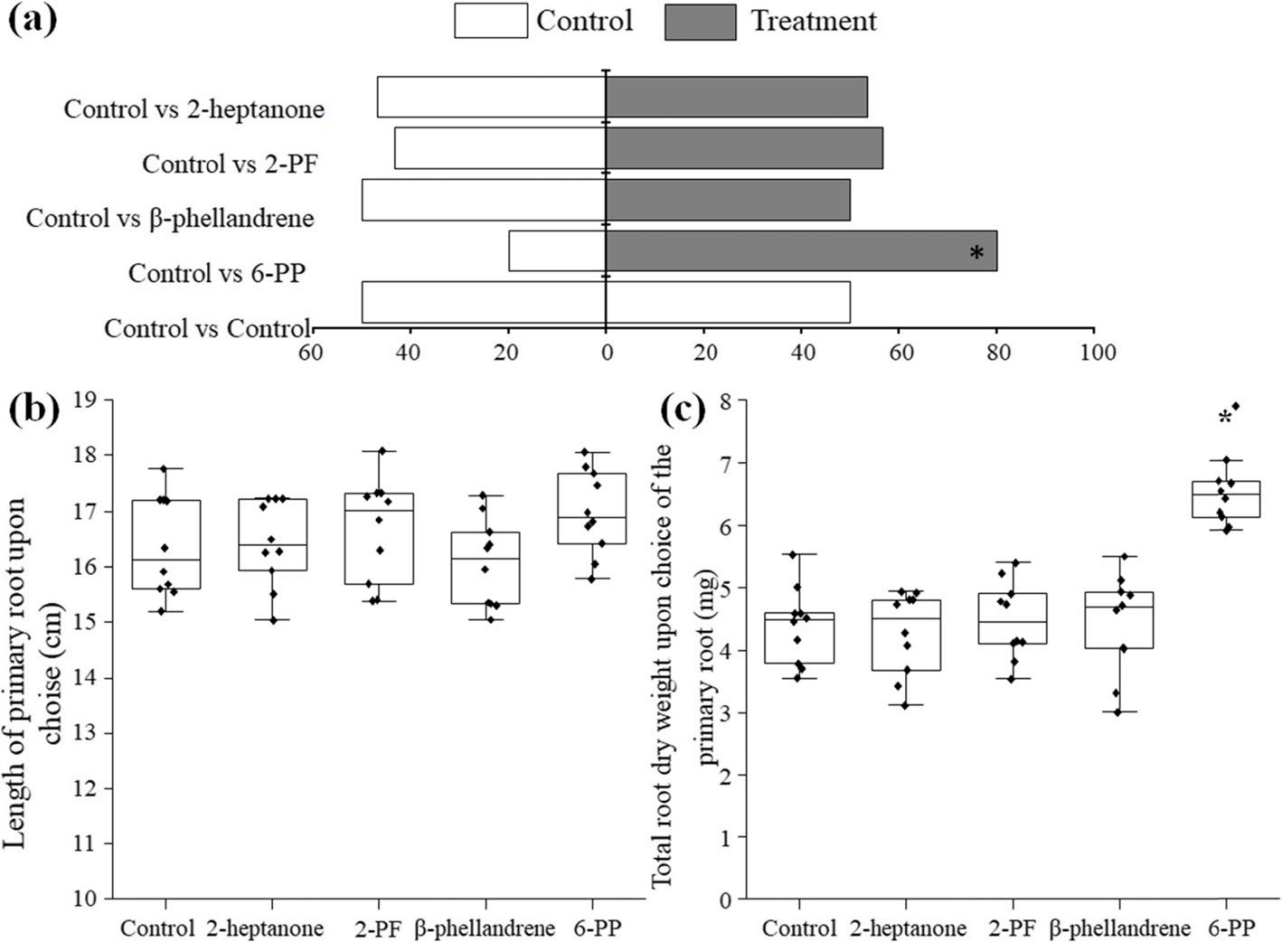
Effects of pure volatile compounds on the root growth of tomato seedlings using modified root Y-tube olfactometers. **a** Percentages of tomato primary roots growing in the control arm or the treatment arm 14 days after sowing. Control versus control was used as a negative control. Twenty root olfactometer replicates were performed. Choices of primary roots were analysed with binomial tests (H_0_ = 0.50). Asterisk indicates pairwise differences between the control and 6-PP choices. **b** Length of the primary root. **c** Total root dry weight. Asterisks (*) indicate significant differences derived from Student *t* test at *P* = 0.05

The effects of the five selected volatiles on the directional growth and growth rate of tomato seedling roots were tested in Y-tube olfactometers. Among these compounds, only 6-PP exhibited a strong ability to influence the direction of primary roots (‘choice’ = 80%; *P* = 0.50) (Fig. 6a). No significant differences in the treatments with 2-heptanone, 2-PF, β-phellandrene, and 3,5,5-trimethyl-cyclohexene were found. Intriguingly, there were no differences in the length of the primary roots between the treatments with any of the tested compounds and the control (Fig. 6b). Among these five compounds, only 6-PP significantly increased the total root dry weight of tomatoes (Fig. 6c).

### Effects of pure VOCs on antifungal activity

The selected pure VOCs were tested for antagonistic activity against the pathogen *F. oxysporum* on PDA plates. The volatile 6-PP produced the strongest inhibitory effect on mycelial growth (Table 3). The EC_50_ of 6-PP against *F. oxysporum* was 5.76 μL mL^−1^ headspace, which is significantly lower than the 18.72 μL mL^−1^ headspace for 2-PF and 34.77 μL mL^−1^ headspace for 2-heptanone.

**Table 3:** Inhibitory effects of volatile compounds on mycelial growth of *Fusarium oxysporum* at 72 h post-inoculation

Chemicals	EC_50_ (μL mL^−1^ headspace)
2-heptanone	34.77
2-pentylfuran	18.72
β-phellandrene	–
3,5,5-trimethyl-cyclohexene	–
6-pentyl-2H-pyran-2-one	5.76

– No mycelial growth inhibition observed

## Discussion

Plant biostimulants formulated with beneficial microorganisms have been developed and applied for the purposes of promoting plant growth, development, and adaptation to abiotic or biotic stress [3]. Among them, *Trichoderma*-based biostimulants have received much attention because of their capacity to control plant diseases caused by certain fungi [28]. Thereafter, *Trichoderma* spp. have been widely studied and are among the fungi most commonly used in agriculture, horticulture, and forestry [2, 4, 29]. However, there is still considerable interest in finding new isolates of *Trichoderma* with more efficient biocontrol or plant biostimulatory activity for the development of more efficient plant biostimulants [30]. In this study, we identified and investigated a coconut-scented *T. atroviride* strain LZ42 with a plant-growth-promoting effect and biocontrol activity. *T. atroviride* LZ42 formulated in talc exhibited a significant control effect on Fusarium wilt of tomatoes in greenhouses and promoted the accumulation of plant biomass and growth, which is consistent with the activity of another *T. atroviride* strain BC0584 [31]. The greenhouse experiments in this study clearly showed the positive effect of *T. atroviride* LZ42 on Fusarium wilt disease control. Our results suggest that *T. atroviride* LZ42 has potential as a biostimulant and/or a biocontrol agent for application during vegetable or other crops growth.

The mechanism through which *Trichoderma* results in biostimulation involves multilevel communication with the root systems of the host plant [32]. Many *Trichoderma* strains are beneficial microorganisms that lead to the activation of defense and developmental responses of the host plant [33–35]. The secondary metabolites released into the rhizosphere by *Trichoderma* strains, including auxins, small peptides, and other active metabolites, can promote root branching and nutrient-uptake capacity, thereby boosting plant growth and yield [12]. *T. atroviride* LZ42 exerted a growth-promoting effect and enhanced root development in the tomato seedlings in this study. This might be associated with the production of indole acetic acid (IAA) and 1-aminocyclopropane-1-carboxylate (ACC) deaminase by *T. atroviride* [21, 36]. However, we focused on the effect of VOCs emitted by *T. atroviride* LZ42 on the root growth of tomato seedlings in this study. It was found that the primary roots of tomato seedlings grew more frequently toward volatiles from *T. atroviride* LZ42 than the control after the root Y-tube olfactometer assays. The root Y-tube olfactometer method had been successfully developed and used to investigate the chemical crosstalk between tomato roots and soil-borne fungi and its impact on root growth [37]. To the best of our knowledge, this is the first study to characterize the effects of VOCs produced by *T. atroviride* on the plant root growth in Y-tube olfactometers.

Fungi produce various mixtures of VOCs that are able to diffuse through the atmosphere and soils [19]. Fungal VOCs are considered important signals for fungi in their natural environments [38]. Fungi use their VOCs to interact with plants, arthropods, bacteria, and other fungi in various habitats [39]. In particular, the roles of VOCs produced by *Trichoderma* spp. in stimulating plant growth and suppressing the growth of plant pathogens have been proven [21]. Our results demonstrate the ability of *T. atroviride* LZ42 to produce volatile antifungal compounds, showing that this is an important mechanism involved in and responsible for the successful inhibition of *F. oxysporum* and effective control of Fusarium wilt disease in tomato seedlings. The VOCs produced by T. atroviride LZ42 positively directed the growth of the primary roots of tomato seedlings and increased the biomass of the roots. These results are consistent with those for other *T. atroviride* strains such as IMI 206,040 [26] and CBS 01–209 [22].

These volatiles produced by fungi have potential for agricultural application, motivating the search for additional bioactive VOCs from a variety of *Trichoderma* species [40]. Among these VOCs, 6-PP, a polyketide with a characteristic sweet coconut-like aroma, is the most common and well-known compound produced by various *Trichoderma* species [40]. The VOC 6-PP is regarded as a potential volatile chemical with a broad-spectrum ability to modulate both root growth and defense responses [22]. Our results show that 6-PP is the dominant VOC released by *T. atroviride* LZ42 after identification through SPME GC/MS, in agreement with the strong coconut-like scent from the fungus. The results of the current study demonstrate that 6-PP emitted by *T. atroviride* LZ42 is the critical factor in fungal biostimulation and biocontrol. Various evidences from the literatures support this result. Taha et al. (2021) indicated that 6-PP elicited the induction of SAR in tobacco and exhibited antiviral activity against tobacco mosaic virus [41]. Garnica-Vergara et al. (2016) found that 6-PP improved the shoot and root growth and total biomass production of *Arabidopsis thaliana* [26]. The VOC 6-PP was also shown to control several plant pathogenic fungi such as *F. moniliforme* [42], *Rhizoctonia solani* [43], and *Athelia rolfsii* [44]. Pascale et al. (2017) suggested that using natural compounds such as 6-PP produced by *Trichoderma* spp. may be an effective alternative strategy [45].

Manipulating the production of bioactive VOCs to improve the performance of *Trichoderma* products remains a challenge. The production of bioactive VOCs in *Trichoderma* spp. is considered to be strain- and condition-dependent [28, 46]. Flores et al. (2019) reported that 6-PP production by *T. atroviride* was mainly elicited by the metabolites from the plant pathogenic fungus *Rhizoctonia solani* [47]. However, Lee et al. (2016) found that 6-PP was not common to all *Trichoderma* strains [22]. It has also been reported that 6-PP does not exist in the VOC profile of either *T. virens* strain G-41 or *T. harzianum* strain T-22 [25]. Though the VOCs produced by *Trichoderma* are much more complex than previously considered, their chemical diversity and communication roles in plant–fungus cross-kingdom signaling are still of interest.

## Conclusion

In this study, we identified the plant biostimulatory and biocontrol activity of *T. atroviride* strain LZ42 on tomato seedlings, which were manifested in plant root growth stimulation and the suppression of disease symptoms. The VOCs produced by *T. atroviride* LZ42, especially 6-PP, are involved in its biostimulatory and biocontrol activities. The results from our study reveal that VOCs emitted by *T. atroviride* LZ42 mediate directed root growth, improve plant growth, and suppress Fusarium wilt disease in tomato plants.

## Methods

### *Trichoderma* and plant pathogens

The antagonistic *Trichoderma* strain LZ42 was previously isolated from *G. lucidum* cultivated soil in Lanju (N27^o^57′55’’, E119^o^02′31’’), Longquan County of China in September, 2015 [27]. The plant pathogen *Fusarium oxysporum* f. sp. *lycopersici* K34 used in this study was obtained from the National and Provincial Joint Engineering Laboratory of Biopesticide Preparation, Zhejiang A&F University, China. All the fungi were grown on PDA medium at 25 °C and restreaked after one month.

### Plant material and growth conditions

Tomato seeds (ZheZa 806, a hybrid variety from a local variety ZheHong 20 and Manapal Tm-2nv) provided by Zhejiang Academy of Agricultural Sciences of China were surface-sterilized with 2% sodium hypochlorite (NaOCl) for 5 min, rinsed four times for 5 min in deionized sterilized water, and sown in foam pots containing a mixture of perlite, peat, and vermiculite (1:1:1 v/v/v). The plants were grown at a maximum temperature of 30 °C (day) and minimum of 21 °C (night) at a relative humidity of 60–85% under daylight conditions in a greenhouse.

### Morphological observation

The *Trichoderma* strain was inoculated on 9 cm-diameter Petri dishes containing PDA and cornmeal dextrose agar (CMD, Oxoid, Hants, U.K.) at 26 °C. The morphological characteristics of the colonies were recorded. Microscopic morphological observations were performed with a Motic M200 microscope (Motic, China) and a scanning electron microscope (Phenom Pro, the Netherlands).

### Molecular identification

The DNA of strain LZ42 was extracted using the Ezup Column Fungi Genomic DNA Purification Kit (Sangon Biotech, Shanghai, China) according to the manufacturer’s protocol. DNA fragments of RNA polymerase II subunit B (*rpb2*) and translation elongation factor 1-alpha (*tef1*) were amplified with the primer pairs fRPB2-5f/fRPB2-7cr [48] and EF1–728F/ TEF1LLErev [49], respectively. These obtained sequences were deposited in the GenBank database at the National Center for Biotechnology Information (NCBI), and the accession numbers are listed in Supplementary Table S1. The combined sequence of *rpb2* and *tef1* from strain LZ42 was assessed by phylogenetic analysis for its variability compared to other species of *Trichoderma* in the NCBI using Mega 7.0 [50]. The phylogeny was analyzed by the bootstrap method with 1000 replications and condensed with a cut-off value of 70%.

### Talc formulation of strain LZ42

Talc were used to carry strain LZ42 in the formulation as decribed by Mukherjee et al. (2014) [51]. Mycelial discs (9 mm) of strain LZ42 grown on PDA medium were inoculated in 1000 mL of PD broth and incubated at room temperature for five days. Afterward, the fungal culture was blended thoroughly and mixed with sterile talc powder (XuFeng©, Quanzhou, China) at a ratio of 1:2 (v/w). The mixture was air-dried and ground gently. The mixture was filtered with Subsequently, the formulated strain LZ42 was assessed for its concentration in colony-forming units (CFU) in PDA medium and had a minimum count of 2.0 × 10^7^ CFU g^−1^.

### Greenhouse assessment

After 30 days of sowing, each tomato seedling (each with four euphylla) was gently transplanted to one pot (26 cm wide and 28 cm deep) containing 1 kg of soil or Fusarium-infested soil. Fusarium-infested soil was obtained by mixing 10 mL *F. oxysporum* spore suspension (10^6^ spores mL^−1^) and 1 kg commercial planting soil (YIWEI, Hangzhou, China) as described previously [52]. The soil was sterilized at 121 °C for 30 min and placed at room temperature for cooling for two days prior to use. Seven treatments were applied as follows: (1) NC, uninfested soil; (2) FI, Fusarium-infested soil; (3) MC, uninfested soil amended with 50 g of the talc formulation alone; (4) MT, uninfested soil amended with 50 g of the strain LZ42 formulation; (5) FT, Fusarium-infested soil amended with 50 g of the strain LZ42 formulation; (6) FA, Fusarium-infested soil amended with 50 g of the talc formulation alone; (7) FC, Fusarium-infested soil mixed with carbendazim, 10 mL of 2.0 mg mL^−1^ carbendazim solution (25%, wettable powder, dissolved in water). Twenty tomato plants were used in each treatment. Each experiment was performed independently 3 times. The wilt incidences for tomato plants were calculated based on the wilt symptoms at 50 days post-inoculation (dpi) while the control plants started the flowering stage. The disease incidence is reported as the percentage of diseased plants out of the total number of plants. The control efficiency was calculated by the formula: control efficiency (%) = (*a*-*b*)/*a* × 100, where *a* is the disease incidence under FA treatment and *b* is the disease incidence under FT or FC treatment. For the determination of the dry plant weight, the plants were thoroughly washed with water and dried at 70 °C until reaching a constant weight.

### Root Y-tube olfactormeter assay

As described by Moisan et al. (2021) [37], a Y-tube olfactometer consisting of a Y-shaped plastic tube (Yinzhan, Hangzhou, China) and two arms was used to study the volatile-mediated interactions. We applied some minor modifications for growing tomato seedlings in the olfactometers (Fig. 4a). Prior to inoculation, these equipments were surface-sterilized with 70% ethanol in a flow cabinet, rinsed with sterile water, and dried by evaporation. The Y-shaped plastic tube was filled with a mixture of perlite, peat, and vermiculite (1:1:1, v/v/v) with the two arms pointing downward. Each arm of the olfactometer was connected to an empty tube which was connected to a 50 ml-centrifugal tube containing agar medium with or without *Trichoderma*, then sealed with parafilm. After assembling all the tubes together, sterile seeds were sown on the soil surface on top of the Y-tubes. The primary roots of the germinated tomato seedlings could grow downward into one of the two arms of the Y-tube: either toward the treatment or toward the control. The assembled root olfactometers were buried in a plant nursery bed filled with sand and placed in a greenhouse. One seed was sown per olfactometer, and 30 independent root olfactometers were used in *Trichoderma* versus control treatment. Plants were harvested 20 days after sowing. Replicates with non-germinated seeds or replicates where the control agar medium developed microbial contamination were discarded. The “choice” of the primary roots in the Y-tube olfactormeter were calculated based on their visual location in either arms. The roots were collected and washed to remove soil particles. The lengths of the primary roots were measured, and all the roots were dried at 70 °C and weighed.

### Antagonistic activity assay for volatiles produced by strain LZ42

Sandwiched Petri plates, a setup described by Li et al. (2018) [25], were employed to test the antagonistic activity of the volatiles produced by strain LZ42 against the plant pathogen *F. oxysporum*. After inoculating the pathogen and strain LZ42 on PDA plates, the pathogen plate was placed on top of a *Trichoderma* plate, sealed with three layers of parafilm, and then incubated at 25 °C. A non-inoculated PDA plate was set as the control. The colony diameters were measured 5 days later. Each treatment included three biological replicates and was repeated three times.

### Analysis of the contents of the volatiles produced by strain LZ42

Qualitative assays of the composition of the volatiles produced by strain LZ42 were performed using solid-phase microextraction coupled with gas chromatography tandem mass spectrometry (SPME–GC–MS) analysis [53]. Strain LZ42 was cultured on PDA and incubated at 25 °C for 5 days. SPME inserted into the injection port of a GC 2010 gas chromatograph was used to collect the volatiles according to the method previously described by Wu et al. (2020) [54]. SPME fiber was exposed to the vapor phase above strain LZ42 for 45 min in a culture tube. Then, the adsorbent fiber was inserted into the injection port of a Hewlett Packard 7890GC/5975MSD gas chromatograph (Agilent Technologies, USA) equipped with an HP-5MS capillary column. Purified helium was used as the carrier gas with a flow rate of 1 mL/min in split–splitless mode. The column temperature was programmed as follows: an initial temperature of 40 °C for 2 min, increased at a rate of 4 °C min^−1^ to 180 °C, and tehen increased to 250 °C at a rate of 5 °C min^−1^, before being maintained at 250 °C for 6 min. The mass spectrometer was operated at unit mass resolution. The derived data were analyzed and identified based on a comparison with the mass spectrum of the GC–MS system in the NIST08.L data bank (National Institute of Standards and Technology). Each experiment was conducted three times.

### Antifungal activity assay for selected pure VOCs

The antifungal activity of each selected pure VOC was tested by fumigation in an isolated container as described in Wu et al. (2020) [54]. Mycelial plugs of *F. oxysporum* (5 mm in diameter) were placed in the center of the PDA. Filter paper (30 mm in diameter) with 1, 2, 4, 8, 16, or 32 μL of each pure VOC (Sigma-Aldrich, MO, USA) was attached inside the cover of the dishes, resulting in 0.01, 0.03, 0.05, 0.11, 0.21, and 0.43 μL mL^−1^ headspace, respectively. Subsequently, the loaded plates were immediately sealed with three layers of parafilm and incubated at 25 °C. The assays for each dose of pure VOCs were conducted with three replicates. Plates with filter paper alone were used as the controls. The percentage inhibition of mycelial growth was calculated according to the formula (*c*–*t*/*c*) × 100, where *t* is the mean diameter of the fungal colonies following the treatment and *c* is the mean value for the control. The EC_50_ values (expressed as μL mL^−1^ headspace) were calculated as the effective concentration that inhibited mycelial growth by 50% in comparison to the control.

### Determination assay of root growth for selected pure VOCs

The root Y-tube olfactormeters described above were used for determination assay of tomato root growth for selected pure VOCs, except that 50 μl of each selected pure VOC instead of *Trichoderma* plates were added into the 50-ml centrifuge tube. The empty centrifuge tube was set as control. One seed was sown per olfactometer, and 30 independent root olfactometers were used in each selected pure VOC versus control treatment. Control versus control was used as a negative control. The “choice” of the primary roots and root growth were determined as described above.

### Statistical Analysis

Tukey’s HSD test was used to assess the differences between means for the antagonistic activity and growth promotion of tomatoes for treatment with *T. atroviride* LZ42 or the fungal VOCs. The “choice” of the primary roots was analyzed with binomial tests, and frequencies of root growth toward fungal volatiles were.

compared between the four fungi using a *χ*2 test (*H*_*0*_ = 0.50). Differences in plant dry weights between the control and treatment were analyzed with Student *t*-tests. The significance was evaluated at *P* = 0.05 in all the tests. The statistical analyses were performed in the IBM SPSS Statistics v.19 program (SPSS, Inc.).

## Supplementary Information

**Additional file 1:** **TableS1.** GenBank accession numbers oftaxa used in phylogenetic analyses.

**Additional file 2:** **Figure S1.** Chromatogram of the volatilesemitted by *Trichoderma atroviride*LZ42 analyzed through SPME GC/MS.

## Data Availability

The DNA sequence data generated and/or analysed during the current study are available in the GenBank nucleotide database (https://www.ncbi.nlm.nih.gov/) under accession numbers MZ375489 and MZ375490. The other datasets used or analysed during the current study are available from the corresponding author on reasonable request.
