# Supplementary material for: Trichoderma atroviride LZ42 releases volatile organic compounds promoting plant growth and suppressing Fusarium wilt disease in tomato seedlings
Source: BMC Microbiol. 2022 Apr 5;22:88. doi: 10.1186/s12866-022-02511-3 (PMC8981656; doi:10.1186/s12866-022-02511-3)
Supplement: Supplementary file 1 — Additional file 1: TableS1. GenBank accession numbers oftaxa used in phylogenetic analyses. [file 12866_2022_2511_MOESM1_ESM.docx]

**Table S1.** GenBank accession numbers of taxa used in phylogenetic analyses.

| Species name | Strain number | GenBank accession number | |
| --- | --- | --- | --- |
|  |  | *rpb2* | *tef1* |
| *T. asperellum* | G.J.S. 90-7 | EU338337 | EU338333 |
| *T. atroviride* | TRS26 | KP009054 | KJ786832 |
|  | CBS 119499=Hypo 326 | FJ860518 | FJ860611 |
|  | LZ42 | MZ375489 | MZ375490 |
| *T. gamsii* | G.J.S. 04-09 | JN133561 | DQ307541 |
| *T. guizhouense* | S628 | KJ665273 | KJ665511 |
| *T. hamatum* | Hypo 648=CBS 132565 | KJ665275 | KJ665514 |
| *T. harzianum* | CBS 226.95* | AF545549 | AF534621 |
|  | TRS55 | KP009121 | KP008803 |
|  | TRS94 | KP009120 | KP008802 |
| *T. koningiopsis* | DIS 172ai=CBS 119067 | FJ442768 | DQ284972 |
| *T. longisporum* | HMAS 248843 | KY687982 | KY688043 |
| *T. paratroviride* | S385=CBS 136489* | KJ665321 | KJ665627 |
| *T. pseudokoningii* | G.J.S. 81-300 | HM182985 | AY937429 |
| *T. reesei* | G.J.S. 00-89 | JN175548 | JN175599 |
| *T. spirale* | TRS111 | KP009182 | KP008963 |
| *T. velutinum* | C.P.K. 298 | KF134794 | KJ665769 |
| *T. virens* | Gli39=CBS 249.59 | AF545558 | AF534631 |
| *T. viride* | CBS 119325 | EU711362 | DQ672615 |
|  | TRS575 | KP009081 | KP008931 |
| *Protocrea farinosa* | MH863119 | EU703935 | EU703889 |
