# Supplementary material for: Trichoderma atroviride LZ42 releases volatile organic compounds promoting plant growth and suppressing Fusarium wilt disease in tomato seedlings
Source: BMC Microbiol. 2022 Apr 5;22:88. doi: 10.1186/s12866-022-02511-3 (PMC8981656; doi:10.1186/s12866-022-02511-3)
Supplement: Supplementary file 2 — Additional file 2: Figure S1. Chromatogram of the volatilesemitted by Trichoderma atrovirideLZ42 analyzed through SPME GC/MS. [file 12866_2022_2511_MOESM2_ESM.docx]

**
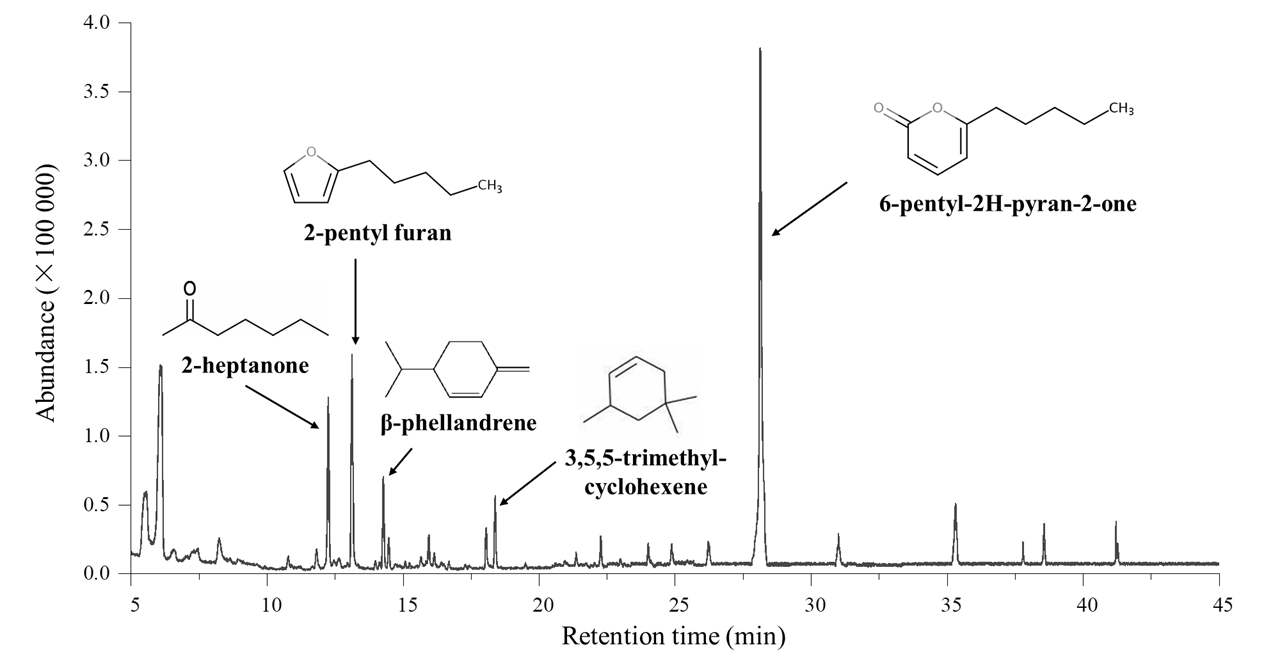
**

**Fig. S1.** Chromatogram of the volatiles emitted by *Trichoderma atroviride* LZ42 analyzed through SPME GC/MS.
